# Basic fibroblast growth factor in the bone microenvironment enhances cell motility and invasion of Ewing's sarcoma family of tumours by activating the FGFR1–PI3K–Rac1 pathway

**DOI:** 10.1038/sj.bjc.6605775

**Published:** 2010-07-06

**Authors:** S Kamura, Y Matsumoto, J-i Fukushi, T Fujiwara, K Iida, Y Okada, Y Iwamoto

**Affiliations:** 1 Department of Orthopaedic Surgery, Graduate School of Medical Sciences, Kyushu University, 3-1-1 Maidashi, Higashi-ku, Fukuoka 812-8582, Japan

**Keywords:** Ewing's sarcoma family of tumours, basic fibroblast growth factor, cell motility, bone microenvironment, PI3K, Rac1

## Abstract

**Background:**

Ewing's sarcoma family of tumours (ESFT) is a malignant small round-cell tumour of the bone and soft tissues. It is characterised by a strong tendency to invade and form metastases. The microenvironment of the bone marrow is a large repository for many growth factors, including the basic fibroblast growth factor (bFGF). However, the role of bFGF in the invasive and metastatic phenotype of ESFT has not been investigated.

**Methods:**

The motility and invasion of ESFT cells were assessed by a wound-healing assay, chemotaxis assay, and invasion assay. The expression and activation of FGF receptors (FGFRs) in ESFT cell lines and clinical samples were detected by RT–PCR, western blotting, and immunohistochemistry. The morphology of ESFT cells was investigated by phase-contrast microscopy and fluorescence staining for actin. Activation of Rac1 was analysed by a pull-down assay.

**Results:**

bFGF strongly induced the motility and invasion of ESFT cells. Furthermore, FGFR1 was found to be expressed and activated in clinical samples of ESFT. Basic FGF-induced cell motility was mediated through the FGFR1–phosphatidylinositol 3-kinase (PI3K)–Rac1 pathway. Conditioned medium from bone marrow stromal cells induced the motility of ESFT cells by activating bFGF/FGFR1 signalling.

**Conclusion:**

The bFGF–FGFR1–PI3K–Rac1 pathway in the bone microenvironment may have a significant role in the invasion and metastasis of ESFT.

Ewing's sarcoma family of tumours (ESFT) is a small round-cell tumour that typically arises in the bones and rarely in the soft tissues of children and adolescents. It contains a characteristic chromosomal translocation, t(11; 22), that is present in 90–95% of tumours. The resulting fusion protein, EWS/FLI1, contains the amino terminus of EWS and the carboxy terminus of FLI1. Ewing's sarcoma family of tumours is a highly metastatic tumour, and patients with metastatic ESFT typically have a poor prognosis (Burchill, 2003; Iwamoto, 2007). Despite new chemotherapeutic regimens, treatments for metastatic ESFT have been largely unsuccessful (Pinkerton *et al*, 2001; Bernstein *et al*, 2006; Miser *et al*, 2007). Therefore, it is important to identify and validate novel therapeutic targets that can prevent the metastasis of ESFT.

The process of tumour cell invasion and metastasis is believed to occur in sequential steps. During tumour progression, subsets of cells change from an immotile to motile state. Motile cells then detach from the primary tumour, enter blood/lymphatic vessels, and seed in distant organs (Sahai, 2007; Nguyen *et al*, 2009). Thus, learning more about the cellular and molecular machinery that controls cell motility will help us understand how tumour cells disseminate and generate metastases. The principles of cell motility were initially investigated in non-neoplastic cells, such as fibroblasts and keratinocytes. Recent studies have also shown that the basic machinery of cell motility is retained in tumour cells, particularly in cancer cells (Friedl and Wolf, 2003; Yamazaki *et al*, 2005). However, the mechanisms that control the motility of ESFT cells have not been clearly described, despite the aggressive nature of these tumour cells.

The bone is a large repository for many growth factors, including transforming growth factor-*β* (TGF-*β*), hepatocyte growth factor (HGF), endothelial growth factor (EGF), insulin-like growth factor (IGF), *β*-platelet-derived growth factor (PDGF), and fibroblast growth factor (FGF), and provides a rich environment for tumour cell growth (Bussard *et al*, 2008). Moreover, growth factors in the bone microenvironment can stimulate the migration of tumour cells by acting as chemoattractant molecules (Mundy *et al*, 1981; Mundy, 2002). Recent studies have begun to elucidate the effects of certain growth factors within the bone microenvironment on the biological phenotype of ESFT. For example, IGF-1 signalling is essential for tumourigenesis (Toretsky *et al*, 1997), maintenance of tumour growth (Scotlandi *et al*, 1998), and ESFT cell migration. In addition, PDGF-BB was shown to enhance the growth and cell migration of ESFT (Uren *et al*, 2003), and PDGF receptors are reportedly activated in clinical ESFT specimens (Bozzi *et al*, 2007).

Basic FGF (bFGF) mediates various cellular events, including proliferation, motility, and differentiation (Powers *et al*, 2000; Bottcher and Niehrs, 2005; Chalkiadaki *et al*, 2009). However, there are several contradictory results on the biological functions of bFGF in ESFT. Basic FGF has been shown to induce the growth arrest and apoptosis of ESFT cells *in vitro* (Sturla *et al*, 2000; Westwood *et al*, 2002). In contrast, it was also shown that bFGF may support the proliferation of ESFT cells and maintain their malignant phenotype by upregulating the EWS/FLI1 protein under serum-free conditions (Girnita *et al*, 2000). Hence, the biological and clinical impact of bFGF signalling in ESFT cells is still unknown. Basic FGF stimulates the cell motility of fibroblasts (Bikfalvi *et al*, 1997) and various types of tumours, such as melanoma, prostate cancer, and glioma (Powers *et al*, 2000; Kwabi-Addo *et al*, 2004; Chalkiadaki *et al*, 2009). However, the impact of bFGF on the motility of sarcoma cells, including ESFT, has not been investigated.

In this study, we showed that bFGF significantly enhanced the motility and invasion of ESFT cells. Remarkably, we found that FGFR1 was activated in clinical samples of ESFT. Next, we determined that bone marrow stromal cells (BMSCs) could be a source of bFGF in the bone microenvironment and may promote a metastatic phenotype in ESFT cells by stimulating cell motility. Finally, we showed that activation of the phosphatidylinositol 3-kinase (PI3K)–Rac1 signalling pathway downstream of FGFR was essential for bFGF-induced cell motility in ESFT.

## Materials and methods

### Cell culture

Human ES cell lines, RD-ES, SK-ES-1, SK-N-MC, and WE68, as well as human osteosarcoma cell lines, MG63, SaOS2, and U2-OS, were obtained from the American Type Culture Collection (Rockville, MD, USA). The human ES cell line WE68 was kindly provided by Dr Frans van Valen (Westfalische-Wilhelms University, Munster, Germany). Both RD-ES and WE68 cells were maintained in RPMI 1640 (Invitrogen, Carlsbad, CA, USA), whereas the other cell lines were cultured in Dulbecco's modified Eagle's medium (DMEM, Invitrogen). Both of these media preparations were supplemented with 10% FBS, 100 mg ml^−1^ penicillin, and 100 mg ml^−1^ streptomycin. The cells were incubated at 37°C in a humidified atmosphere containing 5% CO_2_. Human BMSCs were purchased from Lonza Inc. (Walkersville, MD, USA) and cultured in RPMI 1640 containing 10% FBS at 37°C with 5% CO_2_.

### Reagents

Recombinant human bFGF, IGF-1, PDGF-BB, HGF, EGF, TGF-*β*1, and an anti-FGF basic antibody were purchased from R&D Systems (Minneapolis, MN, USA). SU5402, PD98059, LY294002, U-73122, a Rac1 inhibitor (NSC23766), and rapamycin were purchased from Calbiochem (San Diego, CA, USA). A Rho inhibitor was purchased from Cytoskeleton (Denver, CO, USA). The anti-FGF receptor1 antibody and anti-phospho-FGFR-pY653/654 antibody were purchased from Cell Signaling Technology (Beverly, MA, USA) and Abgent (San Diego, CA, USA), respectively. The anti-FGFR2 antibody was purchased from Novus Biologicals (Littelton, CO, USA), and anti-FGFR3 and anti-FGFR4 antibodies were purchased from Santa Cruz Biotechnology (Santa Cruz, CA, USA).

### Wound-healing assay

Wound-healing assays were performed as described previously (Riou *et al*, 2006). Confluent cell monolayers in 6-well plates were wounded by scraping with a micropipette tip. The cells were washed and then cultured in complete media containing the noted reagents and inhibitors. Time-lapse videomicroscopy was performed to monitor wound repair using the CCM-330F monitoring system (Astec Co., Tokyo, Japan) in a 37°C incubator with 5% CO_2_. The degree of wound closure was assessed in five randomly chosen regions by measuring the distance between the wound edges just after wounding and after 12 h. Cells were stained using the Diff-Quik kit (Sysmex, Hyogo, Japan) as follows: the upper chamber was immersed in a Diff-Quik fixative for 2 min, drained, and immersed sequentially in solution I and solution II for 1 min on a 24-well plate.

### Chemotaxis assay

Transwell migration assays were performed as described previously (Matsumoto *et al*, 2002; Hanada *et al*, 2005) using modified Boyden chambers, which consist of Transwell (Corning Costar, Cambridge, MA, USA) membrane filter inserts with 8 *μ*m pores in 24-well tissue culture plates. After 24 h of serum starvation, the cells (2 × 10^5^ cells) were harvested and re-plated onto the upper chamber of a Transwell filter with 8 *μ*m pores (Corning Costar) that was coated with type I collagen. The chamber was placed in DMEM containing 0.1% FBS and the indicated growth factors, antibodies, and inhibitors. After 12 h of incubation at 37°C in 5% CO_2_, cells on the upper surface of the filter were completely removed by wiping with a moist cotton swab. Cells that had migrated through the filter and adhered to its lower surface were fixed, stained using the Diff-Quik kit (Sysmex), and counted by examining five fields per filter under a microscope. Each assay was performed in triplicate and repeated three times.

### Invasion assay

Invasion assays were performed using the Biocoat Matrigel invasion chamber (BD Biosciences, Bedford, MA, USA) according to the manufacturer's protocol and as described previously (Harimaya *et al*, 2000). After 24 h of serum starvation, cells (2 × 10^5^ cells) were harvested and re-plated onto the upper chamber of a Transwell filter that was placed in DMEM containing 0.1% FBS and the indicated growth factors. After a 24-h incubation at 37°C in 5% CO_2_, the filters were fixed and the number of cells that had migrated was determined as described for the chemotaxis assay.

### RT–PCR and quantitative real-time PCR

The Institutional Review Board of the Kyushu University School of Medicine, Fukuoka, Japan, approved the protocol for obtaining and examining the surgical ESFT specimens. Ewing's sarcoma family of tumours was diagnosed on the basis of histological features. Total RNA from ESFT clinical samples was extracted using an RNeasy kit (Qiagen, Heiden, Germany). Overall, 1 *μ*g of total cellular RNA was used in a reverse transcription reaction with SuperScriptII reverse transcriptase (Invitrogen). The PCR was performed for 30 (GAPDH) or 35 (FGFR1–4 and bFGF) cycles in a final volume of 50 *μ*l with the following primers: GAPDH (forward: 5′-TTACCAAAAGTGGCCCACTA-3′ and reverse: 5′-GAAAGATGGTGAACTATGCC-3′ product size 450 bp), FGFR1 (forward: 5′-AACTGGGATGTGGAGCTGGAAGTGC-3′ and reverse: 5′-AGGTGGTGTCACTGCCCGAGGGGCT-3′ product size 344 bp), FGFR2 (forward: 5′-ATCTCTCAACCAGAAGTGTACG-3′ and reverse: 5′-CTGTGTTGGTCCAGTATGGTGC-3′ product size 349 bp), FGFR3 (forward: 5′-GGGGCCCACTGTCTGGGTCAAG-3′ and reverse: 5′-GTCTTCGTCATCTCCCGAGGAT-3′ product size 202 bp), FGFR4 (forward: 5′-CCTGTTGGGGGTCCTGCTGAGTGTG-3′ and reverse: 5′-CTTGCTGGGGGTAACTGTGCCTATT-3′ product size 419 bp), and bFGF (forward: 5′-GGCTTCTTCCTGCGCATCCA-3′ and reverse: 5′-GCTCTTAGCAGACATTGGAAGA-3′ product size 354 bp). Each PCR cycle consisted of a denaturation step at 95°C for 30 s or 1 min, a primer-annealing step at 60°C (bFGF and GAPDH), 58°C (FGFR1), or 55°C (FGFR2–4) for 30 s, and an extension step at 72°C for 30 s or 1 min, using HotStar Taq DNA polymerase (Qiagen) for hot-start PCR. The PCR products were examined on a 1.5% agarose gel. Real-time PCR was carried out using a LightCycler 1.5 (Roche Diagnotics, Indianapolis, IN, USA) in a final volume of 20 *μ*l containing 10 *μ*l of SYBR Premix Ex Taq (Perfect Real Time; Takara Bio, Shiga, Japan), 0.1 *μ*mol l^−1^ of each primer, and template DNA did not exceed 100 ng per PCR reaction using the conditions as follows: initial denaturation step at 95°C for 5 s, denaturation at 95°C for 10 s, and annealing at 60°C for 30 s for 40 cycles. The following primers were designed as following; FGFR1 (forward: 5′-CTCCTCTTCTGGGCTGTGCT-3′ and reverse: 5′-TGGACCAGGAAGGACTCCAC-3′ product size 119 bp), FGFR2 (forward: 5′-CCGAATGAAGAACACGACCA-3′ and reverse: 5′-TCATGGAGGAGCTGGACTCA-3′ product size 122 bp), FGFR3 (forward: 5′-TCAAGCACGTGGAGGTGAAT-3′ and reverse: 5′-AGCTCCTTGTCGGTGGTGTT-3′ product size 100 bp), FGFR4 (forward: 5′-TGAAGGTGAAGCAGGTGGAG-3′ and reverse: 5′-CCTTCCCTGGGCTAATGTCA-3′ product size 123 bp), and glyceraldehyde-3-phosphate dehydrogenase (GAPDH) (forward: 5′-GGAAGGCCATGCCAGTGAGC-3′ and reverse: 5′-CATTGTGGAAGGGCTCATGA-3′ product size 194 bp). The expression of mRNA was calculated using LightCycler version 3.5 software (Roche Diagnotics). Data were standardised using GAPDH as a housekeeping gene. A negative control was also prepared in triplicate using distilled water instead of a DNA template. The assay was performed in triplicate and was repeated in at least three separate experiments.

### Western blot analysis

Cells were washed twice with ice-cold PBS, scraped, collected in a microcentrifuge tube, and then centrifuged. The cells were lysed by adding lysis buffer (20 mM Tris-HCL (pH7.5), 150 mM NaCl, 1 mM Na_2_EDTA, 1% Triton X-100, 2.5 mM sodium pyrophosphate, 1 mM *β*-glycerophosphate, 1 mM Na_3_VO_4_, 1 *μ*g ml^−1^ leupeptin, and 1 mM PMSF), with a protease inhibitor cocktail (Complete Mini, EDTA-free; Roche Diagnotics). After incubating the cells for 10 min on ice, the cellular debris was pelleted by centrifuging for 10 min at 12 000 **g** and the resulting protein-containing supernatant was transferred into another tube. The protein levels were determined using the Quant-iT assay kit (Invitrogen). The samples were boiled for 5 min, and 20 *μ*g of total protein from each of sample was separated on a 4–12% gradient pre-cast MOPS polyacrylamide gel (Novex, San Diego, CA, USA) and then transferred onto a nitrocellulose membrane. The filter was blocked with TBS containing 5% non-fat dry milk or 5% BSA and 0.1% Tween 20 for 1 h at room temperature. The filter was then incubated overnight with the appropriate primary antibodies at 4°C. After washing the filter, a horseradish peroxidase-conjugated secondary antibody (Santa Cruz Biotechnology) was added and the filter was incubated at room temperature for 1 h. After a final set of washes, the immunoreactivity of the blots was detected using an enhanced chemiluminescence detection system (Amersham, Buckinghamshire, UK).

### Immunohistochemistry

The formalin-fixed and paraffin-embedded tumour specimens were sectioned into 5-*μ*m-thick sections. One section was stained with HE, and the others were immunohistochemically examined using Dako's Envision method. Briefly, the deparaffinised sections were incubated in hydrogen peroxide to abolish endogenous peroxidase activity. Next, the sections were autoclaved to enhance anti-genicity. The sections were incubated in goat serum albumin and then treated with an anti-pFGFR-pY653/654 (RB5787, 1:50; Abgent) antibody or control anti-IgG antibody as the primary antibody for 1 h. Next, the sections were treated with Envision polymers (Dako, Glostrup, Denmark) for 1 h. The reaction products were visualised with diaminobenzidine tetrahydrochloride and counterstained with haematoxylin. After each step, the sections were washed with phosphate-buffered saline.

### Rac1 activation assay

The Rac1 activation assay was performed as described previously (Leung and Bolland, 2007). The RD-ES cells were stimulated with bFGF and pre-treated with or without the indicated inhibitors for 2 h. Next, the cells were lysed with cell lysis buffer for 15 min on ice and then centrifuged at 12 000 **g** for 10 min to collect the supernatants. The samples were incubated with PAK-1-PBD agarose (Cell Biolabs, San Diego, CA, USA) at 4°C for 1 h. The samples were washed three times with cell lysis buffer and then boiled in 4 × LDS sample buffer for 5 min. The boiled samples were subsequently centrifuged at 12 000 **g** for 10 min. Active Rac1 (GTP bound) in the supernatants was detected by western blot using a mouse anti-Rac1 mAb (Cell Biolabs). The optical density was measured for each band using ImageJ software (National Institutes of Health, Bethesda, MD, USA). Rac activity is indicated by the amount of GTP-bound Rac1 normalised to the amount of Rac in whole-cell lysate.

### Analysis of cytoskeletal alterations

The RD-ES cells were seeded on type I collagen-coated coverslips in DMEM with 10% FCS and then incubated for 12 h with bFGF (10 ng ml^−1^). Next, the cells were washed twice with PBS, fixed in 4% paraformaldehyde at room temperature, and permeabilised with 0.1% Triton X-100 in PBS. All preparations were incubated with PBS containing 1% BSA to saturate non-specific binding. Tetramethylrhodamine-5-isothiocyanate (TRITC)-conjugated phalloidin (Sigma, St Louis, MO, USA) was applied for 1 h at room temperature. The coverslips were mounted with Vectashield (Vector Laboratories, Burlingame, CA, USA) using DAPI as a nuclear counterstain and then examined under a fluorescence microscope.

### Statistical analysis

Statistical comparisons were performed using Student's *t*-test. The minimal level of significance was *P*=0.05.

## Results

### bFGF-induced motility and invasion of ESFT cells

**Figure 1:**
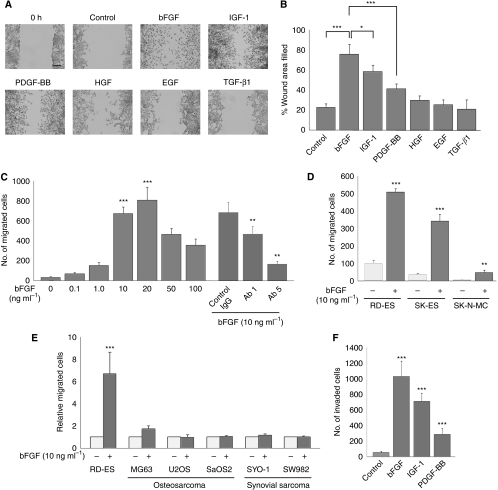
Basic fibroblast growth factor (bFGF)-induced motility and invasion of ESFT cells. (**A** and **B**) An ESFT cell line, RD-ES, was wounded using a rotating silicon tip and treated with growth factors that are typically stored in the bone microenvironment at the indicated concentrations for 12 h: bFGF (10 ng ml^−1^), IGF-1 (20 ng ml^−1^), PDGF-BB (20 ng ml^−1^), HGF (20 ng ml^−1^), EGF (20 ng ml^−1^), and TGF-*β*1(1 ng ml^−1^). In the control experiment, cells were treated with DMEM containing 0.1% serum. (Panel **A**) Representative photographs ( × 10) at 0 and 12 h after wounding. Cells were stained with Diff-Quik kit. Bars: 100 *μ*m. (Panel **B**) The percentage of wound closure corresponds to the distance between the wound edges in five randomly chosen regions. The experiments were repeated at least three times and data are shown as mean±s.d. ^*^P<0.05, ^***^P<0.001. (**C**) The chemotaxis of RD-ES cells was assessed by a chemotaxis assay when various concentrations of bFGF were added to the lower chamber. In addition, RD-ES cells (2 × 10^5^) were either left untreated or pre-treated with the indicated concentrations of an anti-bFGF-neutralising antibody and then subjected to the chemotaxis assay. Data are depicted as mean±s.d. of at least three independent experiments. ^**^P<0.01 *vs* control IgG, ^***^P<0.001 *vs* bFGF 0 ng ml^−1^. (**D**) Effects of bFGF on chemotaxis of various ESFT cell lines, RD-ES, SK-ES-1, and SK-N-MC, were assessed by the chemotaxis assay. Data are depicted as mean±s.d. of at least three independent experiments. ^**^P<0.01, ^***^P<0.001 *vs* an absence of bFGF. (**E**) Effects of bFGF on the chemotaxis of osteosarcoma and synovial sarcoma cell lines were assessed by the chemotaxis assay. Data are depicted as mean±s.d. of at least three independent experiments. ^***^P<0.001 *vs* an absence of bFGF. (**F**) *In vitro* invasion assays were performed in which the indicated growth factors were added to the lower chamber: bFGF (10 ng ml^−1^), IGF-1 (20 ng ml^−1^), PDGF-BB (20 ng ml^−1^). RD-ES cells (2 × 10^5^) were plated onto the upper chamber and incubated for 24 h. The number of cells that migrated across the Matrigel-coated transwell chambers was measured. Experiments were performed in triplicate and repeated at least twice. Data are shown as mean±s.d. ^***^P<0.001 *vs* control.

First, to examine the impact of growth factors that are stored in the bone microenvironment on the motility of ESFT cells, we performed a wound-healing assay and chemotaxis assay. Of the five different growth factors that were tested, bFGF was particularly interesting because it strongly stimulated the chemokinesis of RD-ES cells (% wound closure; control: 22.8±3.8%, bFGF: 76.0±9.9%, IGF-1: 61.0±9.3%, PDGF-BB: 41.3±5.4%, HGF: 29.7±4.7%, EGF: 25.4±5.0%, TGF-*β*: 20.9±9.7%) (Figure 1A and B). Basic FGF enhanced the chemotaxis of RD-ES cells, and an anti-bFGF neutralising antibody suppressed bFGF-induced chemotaxis of RD-ES cells (Figure 1C). In addition, IGF-1 and PDGF-BB were also confirmed to act as chemoattractants for ESFT cells, as was reported previously (Scotlandi *et al*, 1998; Uren *et al*, 2003) (data not shown). The chemokinetic and chemotactic effects of bFGF on RD-ES cells were also supported by a checkerboard analysis (Supplementary Table 1). We further showed that bFGF promoted the motility of other ESFT cell lines, notably SK-ES-1 and SK-N-MC. (Figure 1D). The effects of bFGF on the cell motility of other sarcomas, such as osteosarcomas and synovial sarcomas, were investigated in the chemotaxis assay. Interestingly, the motility of osteosarcoma and synovial sarcoma cell lines did not increase in response to bFGF (Figure 1E). As cell motility is a critical factor for tumour cell invasion, we next examined the effects of bFGF on the invasion of RD-ES cells. We found that bFGF induced the invasion of RD-ES cells, and that the effects of bFGF were more potent than those of IGF-1 and PDGF-BB (Figure 1F). The effect of bFGF in the invasion of other ESFT cell lines, namely SK-ES-1 and SK-N-MC, was observed. (Supplementary Figure 1) On the other hand, bFGF did not enhance the ability of RD-ES cells to attach to the ECM (Supplementary Figure 2A). In addition, bFGF did not stimulate RD-ES cells to produce ECM-degrading enzymes, including matrix metalloproteinases (Supplementary Figure 2B). These findings suggested that bFGF induced the invasion of ESFT cells mainly by increasing cell motility. From these results, we hypothesized that bFGF is one of the critical factors that stimulates the motility and invasion of ESFT cells in the bone microenvironment.

### Expression of FGFRs in ESFT cell lines and clinical samples

**Figure 2:**
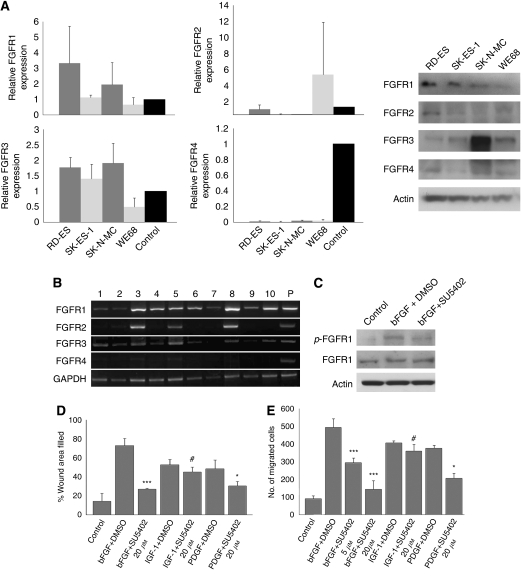
Expression profiles of FGF receptors (FGFRs) in ESFT cell lines and clinical ESFT samples and essential role of FGFR1 in bFGF-induced motility of ESFT cells. (**A**, left panel) Transcript levels of FGFRs, relative to GAPDH, determined by quantitative real-time PCR in ESFT cell lines. Control of FGFR1, 2 and 3; U20S, FGFR4; MG63. (**A**, right panel) Representation of protein status of the FGFRs by western blot in various Ewing's sarcoma cell lines. Actin was used as a loading control. (**B**)The expression profile of ESFT biopsy samples was detected by RT–PCR. GAPDH was used as an internal control. P: positive control. (**C**) RD-ES cells were incubated with bFGF (20 ng ml^−1^) for 20 min, and the total cell lysates were subjected to western blot analysis with anti-FGFR1 and anti-tyrosine-phosphorylated FGFR1 antibodies. In RD-ES cells, bFGF induced the tyrosine phosphorylation of FGFR1, and pre-treating RD-ES cells with a specific inhibitor of FGFR1, SU5402 (20 *μ*M), for 2 h inhibited this phosphorylation. Actin is shown as a loading control. Data are representative of at least three independent trials. (**D** and **E**) The effects of SU5402 on growth factor-induced motility of RD-ES cells were assessed by a wound-healing assay (panel **D**) and a chemotaxis assay (panel **E**). The concentrations of the respective growth factors in the assays were as follows: bFGF (10 ng ml^−1^), IGF-1 (20 ng ml^−1^), and PDGF-BB (20 ng ml^−1^). The growth factors and SU5402 were added into the lower chamber at the same time. In chemotaxis assay, 2 × 10^5^ cells were plated onto the upper chamber. Experiments were performed in triplicate and repeated at least three times. Data are shown as mean±s.d. ^***^P<0.001, ^*^P<0.05 *vs* indicated growth factor with DMSO. #, not significant.

Next, we dissected the molecular mechanism by which bFGF induces the motility of ESFT cells. To examine the expression of the profile of FGFRs in ESFT, we performed a real-time PCR analysis and western blotting analysis for ESFT cell lines, and RT–PCR analysis for ESFT biopsy samples. Consistent with a previous report (Girnita *et al*, 2000), we found that all ESFT cell lines and ESFT biopsy samples expressed FGFR1 (Figure 2A and B). We showed that the moderate-to-high expression of mRNA of FGFR2 was observed in WE68, and that of FGFR3 in all samples. The expression of mRNA of FGFR4 was low in ESFT cells. In contrast, in protein levels, FGFR2 was detected in RD-ES and WE68, FGFR3 in SK-ES-1, SK-N-MC, and WE68, and FGFR4 in RD-ES, SK-N-MC, and WE68. Out of 10 biopsy samples, FGFR2 was detected in 3, FGFR3 in 8, and FGFR4 in 2 (Figure 2B). These results indicated that the expression of FGFRs, particularly that of FGFR1, was a prevalent feature in ESFT. As bFGF binds FGFR1 preferentially (Ornitz *et al*, 1996), we further clarified the role of FGFR1 in ESFT cells. After ligand binding, FGFR dimerises, acquires increased tyrosine kinase activity, and becomes highly phosphorylated on C-terminal cytoplasmic tyrosine residues. When RD-ES cells were treated with bFGF, FGFR1 was tyrosine phosphorylated and a specific inhibitor of FGFR, SU5402, abrogated this tyrosine phosphorylation of FGFR1 (Figure 2C). Similar results were observed in SK-ES-1 cells (Supplementary Figure 3A). To further confirm that bFGF-mediated activation of FGFR1 was required for bFGF-induced cell motility, we examined the effects of SU5402 on bFGF-induced motility of RD-ES cells. The wound-healing assay (Figure 2D) and chemotaxis assay (Figure 2E) both showed that bFGF-induced motility of RD-ES cells was significantly suppressed by SU5402, confirming that the receptor kinase activity of FGFR was required for bFGF-induced motility of RD-ES cells. As SU5402 only weakly inhibits tyrosine phosphorylation of the PDGF receptor, PDGF-BB-induced chemotaxis, but not chemokinesis, was also inhibited (Figure 2D and E). However, IGF-1-induced cell motility was not inhibited by SU5402 (Figure 2D and E). Similar results were observed in SK-ES-1 cells (Supplementary Figure 3B and C).

### Presence of active FGFR1 signalling in clinical samples of ESFT

**Figure 3:**
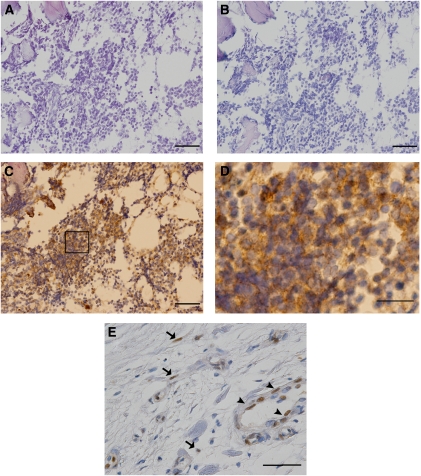
Expression of tyrosine-phosphorylated FGFR1 in clinical ESFT samples. (**A**) A histological section obtained from an ESFT patient was stained with haematoxylin and eosin. (**B**) There was no background staining when a serial section from the same patient as in panel **A** was stained with the control anti-IgG antibody. (**C** and **D**) An anti-tyrosine-phosphorylated FGFR1 antibody induced strong positive staining on the plasma membrane of tumour cells in a serial section from the same patient as in panel **A**. (Panel **D**) Magnification of the inset in panel **C**. (**E**) Normal bone tissue was immunostained with the anti-tyrosine-phosphorylated FGFR1 antibody. Tyrosine-phosphorylated FGFR1 was only observed in endothelial cells (arrowheads) and in a subset of stromal cells (arrows). Scale bars; 50 *μ*m in panels **A**–**C** and 20 *μ*m in panels **D** and **E**.

To confirm that FGFR1 activation in the human bone microenvironment is important in ESFT, we next investigated the expression of tyrosine-phosphorylated FGFR1 in ESFT biopsy samples by immunohistochemistry. Remarkably, we found that seven out of nine (77.8%) ESFT biopsy samples had moderate-to-high levels of phosphorylated FGFR1, whereas serial sections that were stained with the control anti-IgG Ab showed no non-specific signalling (Figure 3C and D). We then analysed the pattern of FGFR1 expression in tissue sections of the normal bone marrow and found ubiquitous expression of FGFR1 in the specimens (data not shown). In contrast, the expression of tyrosine-phosphorylated FGFR1, in most cases, was only detected on the vascular endothelium and the subset of stromal cells within the bone marrow (Figure 3E). Thus, we considered, that FGFR1s are hyper-phosphorylated in Ewing's sarcoma cells. Taken together, these results strongly showed that functional FGFR1 signalling occurs in the human bone microenvironment in ESFTs.

### BMSCs expressed bFGF, and conditioned BMSC medium induced the motility of ESFT cells by activating bFGF/FGFR1 signalling

**Figure 4:**
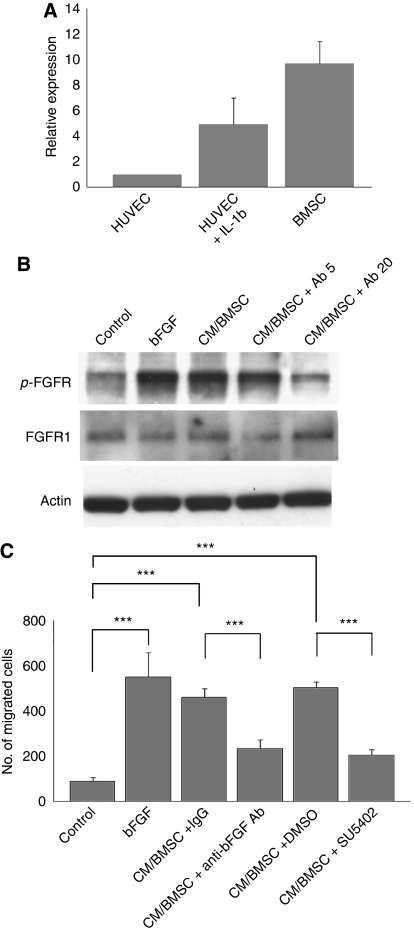
Bone marrow stromal cells (BMSCs) expressed bFGF and conditioned medium from BMSC (CM/BMSC) induced the motility of ESFT cells by stimulating bFGF/FGFR1 signalling. (**A**) bFGF expression in BMSC was assessed by real-time PCR. IL-1*β*-treated human umbilical vein endothelial cells (HUVECs) were used as a positive control. (**B**) CM/BMSC induced the tyrosine phosphorylation of FGFR1 in RD-ES cells. CM-BMSC was harvested from BMSCs that were cultured in a 24-well plate for 72 h. Treating RD-ES cells with CM/BMSC resulted in the tyrosine phosphorylation of FGFR1, and this phosphorylation was reduced by 2 h pre-treating with an anti-bFGF neutralising antibody (5 or 20 *μ*g ml^−1^) to the CM/BMSC. Actin was used as a loading control. Data are representative of at least three independent trials. (**C**) The chemotaxis of RD-ES cells in response to CM/BMSC was assessed by the chemotaxis assay. RD-ES cells (2 × 10^5^) were plated onto the upper chamber. CM/BMSC enhanced the chemotaxis of RD-ES cells, whereas an anti-bFGF-neutralizing antibody (5 *μ*g ml^−1^) and SU5402 (20 *μ*M) impaired the chemotaxis of RD-ES cells towards CM/BMSC. Data are depicted as mean±s.d. of at least three independent experiments. ^***^P<0.001.

The microenvironment of the local host tissue can actively participate in the progression and metastasis of tumour cells. For example, at the boundary between tumour cells and normal host tissues, such as stromal cells, these cells exchange several factors that modify the local ECM, promote proliferation and survival, and stimulate cell motility. It was reported that BMSCs produce bFGF and that their ECM can serve as a reservoir for this growth factor (Brunner *et al*, 1993). Thus, we hypothesized that BMSCs are a possible source of bFGF in the bone microenvironment. To examine this hypothesis, we first confirmed that bFGF was expressed in human BMSCs by examining bFGF mRNA levels by real-time PCR (Figure 4A). We then cultured BMSCs for 72 h and collected the conditioned medium (CM/BMSC). Remarkably, CM/BMSC enhanced the tyrosine phosphorylation of FGFR1 in RD-ES cells, and a specific anti-bFGF-neutralizing antibody reduced this CM/BMSC-induced phosphorylation (Figure 4B). Next, we tested the effects of CM/BMSC on the motility of RD-ES cells. The CM/BMSC significantly induced chemotaxis of RD-ES cells and neutralizing antibodies to both bFGF and SU5402 reduced the CM/BMSC-induced chemotaxis of RD-ES cells (Figure 4C). To examine whether this is dependent on cell type, we also studied the effect of CM/BMSC in SK-ES-1, and basically similar results were observed in SK-ES-1 (Supplementary Figure 4A and B). The chemokinesis of RD-ES cells was also slightly induced by CM/BMSC (data not shown). These results strongly suggested that CM/BMSC enhanced the motility of ESFT cells by activating bFGF/FGFR1 signalling.

### The PI3K pathway was involved in bFGF-induced motility of ESFT cells

**Figure 5:**
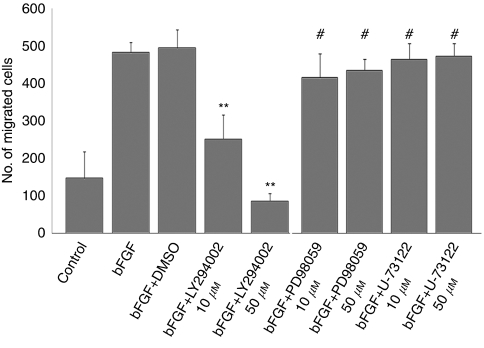
Effects of pharmacological inactivation of the PI3K, MAPK, and PLC-*γ* pathways on bFGF-induced chemotaxis of ESFT cells. LY294002 (PI3K inhibitor), PD98059 (MAPK inhibitor), and U-73122 (PLC-*γ* inhibitor) were used. The effects of each inhibitor on bFGF-induced chemotaxis of RD-ES cells were assessed using a chemotaxis assay with bFGF (10 ng ml^−1^). LY294002 significantly inhibited bFGF-induced chemotaxis, whereas PD98059 and U-73122 had no effect. In all, 2 × 10^5^ cells were plated onto the upper chamber. bFGF and each inhibitor were added at the same time. Experiments were performed in triplicate and repeated at least three times. Data are shown as mean±s.d. ^**^P<0.01 *vs* bFGF with DMSO, #, not significant.

The binding of bFGF to FGFRs and to heparin sulphate proteoglycans (HSPGs) leads to the formation of a ternary complex between FGF, FGFR, and HSPGs. This complex provides a platform to recruit and activate downstream signalling modules, including PI3K, mitogen-activated protein kinase (MAPK) cascade, and phospholipase C (PLC)*γ* (Dailey *et al*, 2005). To identify the dominant signalling pathway downstream of FGFRs that was required for bFGF-induced cell motility, we used various pharmacological inhibitors, including LY294002 for PI3K, PD98059 for MEK/ERK, and U-73122 for PLC*γ*. LY294002, but not PD98059 and U-73122, remarkably attenuated bFGF-induced chemotaxis of RD-ES cells (Figure 5). Similar results were observed in SK-ES-1 cells (Supplementary Figure 5). A wound-healing assay confirmed that LY294002 also significantly suppressed bFGF-induced chemokinesis (% wound closure; control: 14.7±8.9%, bFGF+DMSO: 74.9±8.0%, LY294002: 9.3±3.5%). These results suggested that PI3K activation downstream of FGFRs was indispensable for the bFGF-induced motility of ESFT cells.

### bFGF induced significant morphological alterations of ESFT cells

**Figure 6:**
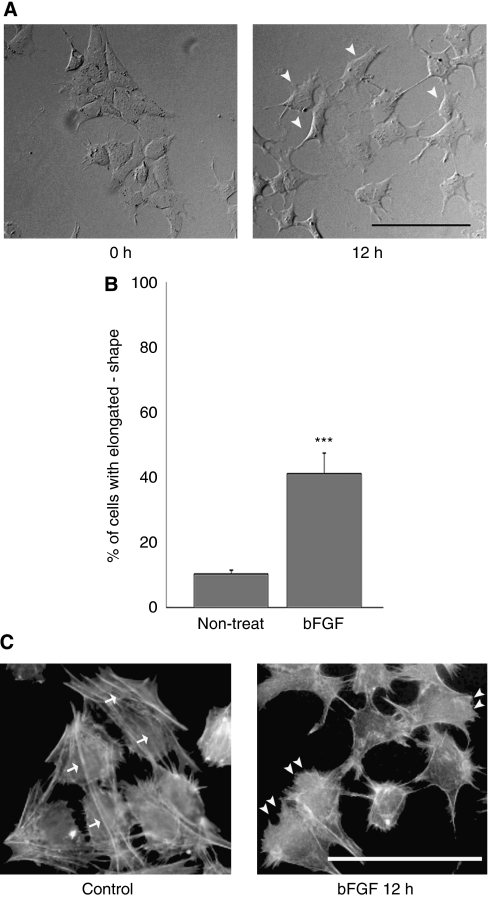
bFGF-mediated morphological alterations of RD-ES cells. (**A**) After RD-ES cells were seeded to 35 mm dishes and incubated for 12 h, bFGF (10 ng ml^−1^) was added to the medium. Representative photographs of phase-contrast microscopy ( × 10) were taken at 0 h (left panel) and 12 h (right panel) after bFGF stimulation. The cell morphology changed from a round shape to a polarised and elongated shape upon bFGF treatment (arrowheads in right panel) Scale bar: 100 *μ*m. (**B**) The percentage of cells with an elongated shape among the total cell population was quantified. Cells were counted in five fields per dish and assayed in triplicate for each condition. Data are depicted as mean±s.d. ^***^P<0.001. (**C**) RD-ES cells were seeded and incubated with bFGF (10 ng ml^−1^) for 12 h. The cells were fixed and stained with TRITC-conjugated phalloidin. Non-treated, control RD-ES cells had a polygonal shape and organised stress fibres (arrows in left panel). In contrast, bFGF treatment induced the organisation of cortical actin with a remarkable degree of lamellipodia formation (arrowheads in right panel). Scale bar: 50 *μ*m.

Recently, PI3K activation was found to be essential for cellular polarisation and elongation, which are the first steps in cell motility (Barber and Welch, 2006). Thus, we studied the morphological alterations of RD-ES cells upon bFGF treatment. We recorded cellular movements and alterations by time-lapse videomicroscopy. We observed that bFGF treatment resulted in cell scattering and an altered cellular morphology from a round shape to a polarised and elongated shape (arrowheads in Figure 6A, right panel). The percentage of cells exhibiting an elongated shape was visualised and quantitated using phase-contrast microscopy. As shown in Figure 6B, bFGF stimulated a two-fold increase in the percentage of elongated cells (treated with bFGF: 38.3±5.2%, control: 11.9±1.2% *P*<0.001). Next, we examined cytoskeletal alterations in RD-ES cells by phalloidin staining. Non-treated RD-ES cells (control) had a polygonal shape with organised stress fibres (arrows in Figure 6C, left panel). In contrast, treating RD-ES cells with bFGF for 8 h resulted in the organisation of cortical actin with a remarkable degree of lamellipodia formation (arrowheads in Figure 6C, right panel). Similar cytoskeletal alteration by bFGF treatment was observed in SK-ES-1 cells (Supplementary Figure 6).

### Rac1 was essential for bFGF-induced motility of ESFT cells

**Figure 7:**
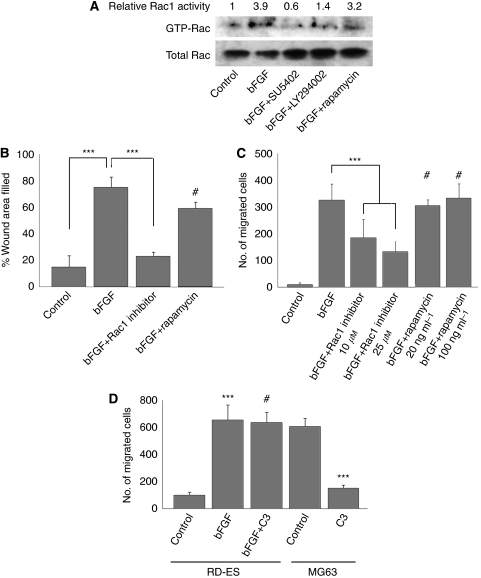
Rac1 was essential for bFGF-induced motility of ESFT cells. (**A**) Rac1 activation in RD-ES cells was assessed using glutathione *S*-transferase (GST)-p21-binding domain (PBD) beads to isolate GTP-bound Rac1. RD-ES cells were stimulated by bFGF (10 ng ml^−1^) with or without pre-treatment with SU5402 (20 *μ*M), LY294002 (50 *μ*M), or rapamycin (50 ng ml^−1^) for 2 h. bFGF increased the activation of Rac1, whereas SU5402 and LY294002 inhibited Rac1 activation. In contrast, rapamycin did not affect bFGF-induced Rac1 activation. Rac1 activity was indicated by the amount of GTP-bound Rac1 normalised to the amount of total Rac1 in whole-cell lysate. The mean value of Rac1 activity relative to serum-starved control cells was indicated as relative Rac activity. (**B** and **C**) The effects of a Rac1 inhibitor and rapamycin on the bFGF-induced motility of RD-ES cells were investigated in a wound-healing assay (panel **B**) and chemotaxis assay (panel **C**). The bFGF-induced motility of RD-ES cells was inhibited by the Rac1 inhibitor (20 *μ*M) but not by rapamycin (20 ng ml^−1^). Data are depicted as mean±s.d. of at least three independent experiments. ^***^P<0.001, #, not significant. (**D**) Effects of a specific Rho inhibitor, C3, on bFGF-induced chemotaxis. bFGF-induced chemotaxis of RD-ES cells was not reduced by C3 (7.5 *μ*g ml^−1^). In contrast, chemotaxis of a human osteosarcoma cell line, MG-63, in response to serum was inhibited by C3. In chemotaxis assay, 2 × 10^5^ cells were plated onto the upper chamber. Data are depicted as mean±s.d. of at least three independent experiments. ^***^P<0.001 *vs* control. #, not significant.

The Rho family of small GTP-binding proteins includes key signalling molecules that regulate cell polarisation and motility (Heasman and Ridley, 2008). Rac1, a small GTP-binding protein, acts as a downstream modulator of PI3K-induced cell polarisation and lamellipodia formation in leukocytes (Inoue and Meyer, 2008; Bosco *et al*, 2009). Therefore, we considered that Rac1 might be involved in bFGF-induced motility in ESFT cells. To test this hypothesis, we first determined the kinetics of Rac1 activation in RD-ES cells after bFGF stimulation. As shown in Figure 7A, Rac1 was activated upon bFGF stimulation. We repeated the experiments at least three times and quantified ‘the mean activity of Rac activation’ by densitometric analysis (Figure 7A). This activation was suppressed by SU5402. Importantly, LY294002 inhibited Rac1 activation upon bFGF stimulation, indicating that Rac1 acts as a downstream modulator of the FGFR/PI3K pathway (Figure 7A). We then showed that pharmacologically inhibiting Rac1 abrogated bFGF-induced cell motility (Figure 7B and C). The Akt/mTOR pathway is another dominant downstream modulator of PI3K, and the Akt/mTOR pathway is involved in IGF-1-induced cell motility and cytoskeletal alterations in ESFT cells (Liu *et al*, 2008). Therefore, we also studied the role of the Akt/mTOR pathway in bFGF-induced motility in RD-ES cells. Rapamycin, a selective inhibitor of the Akt/mTOR pathway, did not reduce Rac1 activation after bFGF treatment (Figure 7A), indicating that the Akt/mTOR pathway had no effect on bFGF-induced Rac1 activation. In addition, rapamycin treatment did not reduce bFGF-induced cell motility (Figure 7B and C). These results implied that the Akt/mTOR pathway may not be involved in the bFGF-induced motility of ESFT cells. Similar results were observed in SK-ES-1 cells (Supplementary Figure 7A–C). Rho is another prototypical small GTP-binding protein in the Rho family that contributes to cell movement by generating actomyosin contractile forces (Amano *et al*, 1997). In sarcomas, RhoA regulates the motility of osteosarcoma cell lines (Matsumoto *et al*, 2001). Thus, we assessed the effects of RhoA on bFGF-induced cell motility using a selective Rho inhibitor, C3 transferase. As shown in Figure 7D, C3 did not significantly inhibit the bFGF-induced motility of RD-ES cells, whereas the locomotion of MG63 cells, an osteosarcoma cell line, was significantly inhibited by C3 treatment. Similar results were observed in SK-ES-1 cells (Supplementary Figure 7C). Taken together, these results suggested that Rac1, but not AKT/mTOR or Rho, is a downstream modulator of PI3K and an essential factor in the bFGF-induced motility of ESFT cells.

## Discussion

Ewing's sarcoma family of tumours is a bone tumour that is characterised by a distinct metastatic pattern that involves the lung and bone. In addition, ESFT cell lines metastasize to the bone at a high frequency in immunodeficient mice (Scotlandi *et al*, 2000; Vormoor *et al*, 2001), showing that the bone microenvironment is supportive for ESFT growth and metastasis. In this study, we identified the factor that enhances the motility of ESFT cells in the bone microenvironment. Our results showed that bFGF, a growth factor that is abundant in the bone microenvironment (Brunner *et al*, 1993), strongly induced the motility of ESFT cells. Although bFGF has been shown to promote the *in vitro* motility of tumour cells, such as prostate cancer cells and melanoma cells (Kwabi-Addo *et al*, 2004; Chalkiadaki *et al*, 2009), this is the first report that describes the effects of bFGF on the motility of ESFT cells. Furthermore, the effects of bFGF were specific because bFGF did not stimulate another highly motile osteosarcoma or synovial sarcoma cells.

Cell motility can be further distinguished as chemotaxis or chemokinesis based on the cellular response to soluble factors (Kohn *et al*, 1990). Chemotaxis is cell motility that is directed towards diffusible factors and has an important role in homing mechanisms (Muller *et al*, 2001). On the other hand, chemokinesis is stochastic movement in response to soluble factors in the absence of a gradient. Chemokinesis may have a role in initiating the random migration of tumour cells out of the primary tumour site (Tchou-Wong *et al*, 2006). In this study, we determined that ESFT cells exhibited a certain degree of chemokinesis and chemotaxis in response to bFGF. As discussed in the previous paragraph, the bone marrow is surrounded by BMSCs and continuously bathed in bFGF secreted by BMSCs. Therefore, bFGF concentration gradients are not likely to be established in this environment. Therefore, the migration of ESFT cells within the bone marrow may be due to chemokinesis, rather than chemotaxis. In contrast, after entrance into blood vessels, the chemotactic properties of bFGF may have a significant role in the bone metastasis of ESFT cells. Therefore, under isotropic conditions, bFGF may enhance the chemokinesis of ESFT cells, but in the presence of a strong gradient, bFGF may act as a potent chemoattractant. These properties of bFGF may be equally important in the invasive and metastatic processes of ESFT.

To date, few studies have investigated the role of growth factor pathways in ESFT and most studies have focused on IGFs and IGFRs (Scotlandi *et al*, 1998; Herrero-Martin *et al*, 2009). Thus, the biological and clinical impact of FGFR expression in ESFT remains obscure. Among the four different types of FGFRs, FGFR1 and FGFR2 have been specifically linked to tumour cells (Klint and Claesson-Welsh, 1999; Korc and Friesel, 2009), and bFGF binds to FGFR1 (Ornitz *et al*, 1996). In this study, we detected FGFR1 in ESFT cell lines, as reported previously (Girnita *et al*, 2000). The expression of other FGFRs was also detected in several cell lines. We also showed that FGFR1 is expressed in all of the examined ESFT clinical samples. Moreover, we detected the tyrosine-phosphorylated, active forms of FGFRs in clinical samples of ESFT, which provided evidence that intact and functional FGFR signalling occurs in ESFT. Taken together, our data suggest that the expression and activation of FGFR are common features of ESFT. In particular, FGFR1 may have a dominant role in inducing bFGF signalling and stimulating the motility of ESFT cells. However, the roles of other FGFRs in ESFT should also be further investigated.

The importance of the interaction between tumour cells and their microenvironment is increasingly emphasised (Liotta and Kohn, 2001; Joyce and Pollard, 2009). The monolayers of BMSCs have been shown to secrete bFGF into the conditioned medium (Hotfilder *et al*, 2005). Hence, the interplay between ESFT cells and BMSCs is an interesting issue that should be examined further. As expected, the conditioned medium of BMSC (CM/BMSC) remarkably enhanced the motility of ESFT cells. This effect could be partially attributed to bFGF in CM/BMSC because CM/BMSC induced the activation of FGFR1 in ESFT cells, and the addition of a neutralising antibody or SU5402 to the CM/BMSC reduced this CM/BMSC-induced motility of ESFT cells. Meanwhile, ESFT cell lines expressed bFGF2 at both the mRNA and the protein levels (Westwood *et al*, 2002). However, it was reported that bFGF could not be detected in the conditioned medium of ESFT cell lines (Hotfilder *et al*, 2005). Consistent with this finding, we also could not detect FGFR activation under standard culture conditions, indicating that autocrine bFGF/FGFR1 signalling is not constant in ESFT cells. Taken together, these data suggest that bFGF is secreted into the bone microenvironment by BMSCs where it exerts effects upon ESFT cells through a paracrine mechanism.

Further clarification of the downstream effectors in bFGF/FGFR signal transduction may give important clues that will expand our understanding of the mechanisms that promote the metastasis of ESFT cells. Although bFGF/FGFR-stimulated cell proliferation has been well investigated (Yoshida *et al*, 1996), the signalling pathways that promote cell motility downstream of bFGF/FGFR1 are not as well defined. In corneal epithelial cells, bFGF induces a change in cell morphology from a polygonal to fibroblastic shape and a reorganisation of the actin cytoskeleton through PI3K (Lee and Kay, 2003). Consistent with these findings, we showed that bFGF activates the PI3K pathway in ESFT cells, which reorganises the actin cytoskeleton and alters the morphology of ESFT cells from a polygonal to an elongated shape. In addition, our data suggest that these processes are responsible for the bFGF-induced motility of ESFT cells. Recently, a direct and/or indirect link of the activation of PI3K and Rho GTPases, including Rac1, has been shown (Welch *et al*, 2003), and we indicated that PI3K enhanced Rac1 activation in response to bFGF. Our data also suggested that the integrated signalling downstream of bFGF/FGFR1 that increases the motility of ESFT cells was independent of the cellular pathways that induce proliferation (MAPK) and survival (Akt/mTOR), which are also stimulated by bFGF/FGFR in certain cell lines, including ESFT (Mateo-Lozano *et al*, 2003; Williamson *et al*, 2004; Hotfilder *et al*, 2005).

Cell motility requires tight coordination and organisation of the actin cytoskeleton, which is controlled by members of the Rho family of small GTPases, including Rho, Rac, and Cdc42 (Raftopoulou and Hall, 2004). Recently, the cross-talk among members of the Rho GTPase family has been investigated. For instance, several studies have shown that Rac and Rho have antagonistic activities (Sander *et al*, 1999) and that Rac and Cdc42 can be coincidentally activated (Kurokawa *et al*, 2004). In corneal epithelial cells, simultaneous Rac activation and Rho inhibition are necessary to alter the cytoskeleton and induce subsequent cell motility upon bFGF treatment (Lee and Kay, 2003). Meanwhile, C3, a specific Rho inhibitor, suppresses bFGF-stimulated cell motility in fibroblasts (Abe *et al*, 2007). Nonetheless, in this study, we showed that Rac1 mediated the bFGF-induced motility of ESFT cells. In contrast, C3 did not attenuate bFGF-induced cell motility, indicating that Rho, unlike Rac, is dispensable for the bFGF-induced motility of ESFT cells. In addition, we found that non-treated Ewing's sarcoma cell lines showed almost no activation of cdc42 and cdc42 was not activated upon bFGF stimulation (data not shown). These findings imply that the effects of bFGF on the cross-talk between the Rho GTPase family members may depend on the context of the cells.

In conclusion, we showed that bFGF-induced FGFR1/PI3K/Rac1 activation was essential for bFGF-induced motility of ESFT cells. We also showed that bFGF/FGFR1 signalling was active in clinical ESFT samples and that BMSCs may be a source of bFGF. These findings also indicate that this signalling pathway may help identify molecular targets that can be used as potential therapeutics to prevent metastases and to improve the survival of ES patients.
