# Supplementary figures and images for: Basic fibroblast growth factor in the bone microenvironment enhances cell motility and invasion of Ewing's sarcoma family of tumours by activating the FGFR1–PI3K–Rac1 pathway
Source: Br J Cancer. 2010 Jul 6;103(3):370–81. doi: 10.1038/sj.bjc.6605775 (PMC2920026; doi:10.1038/sj.bjc.6605775)

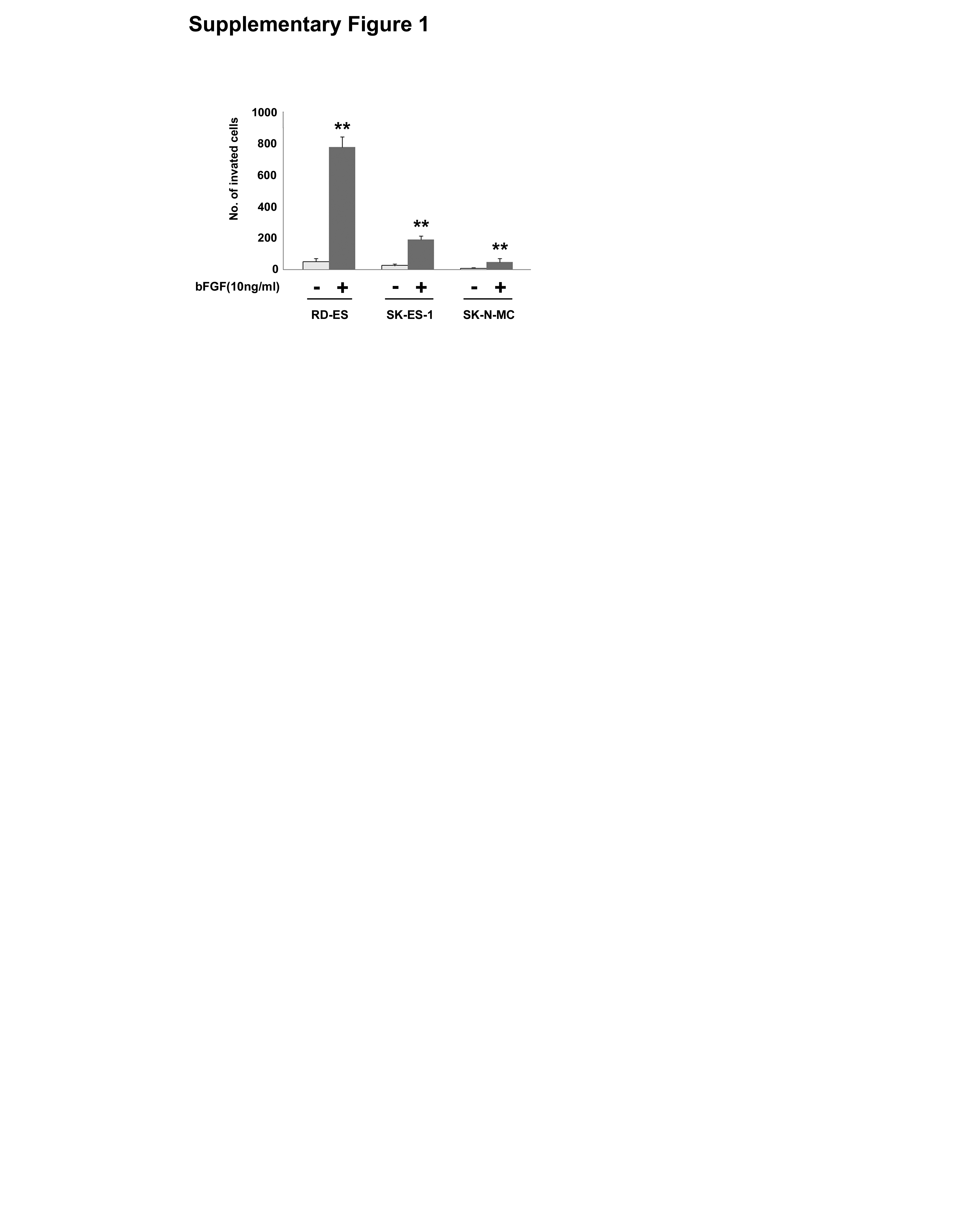

Supplement: Supplementary Figure 1 [file 6605775x1.tif]

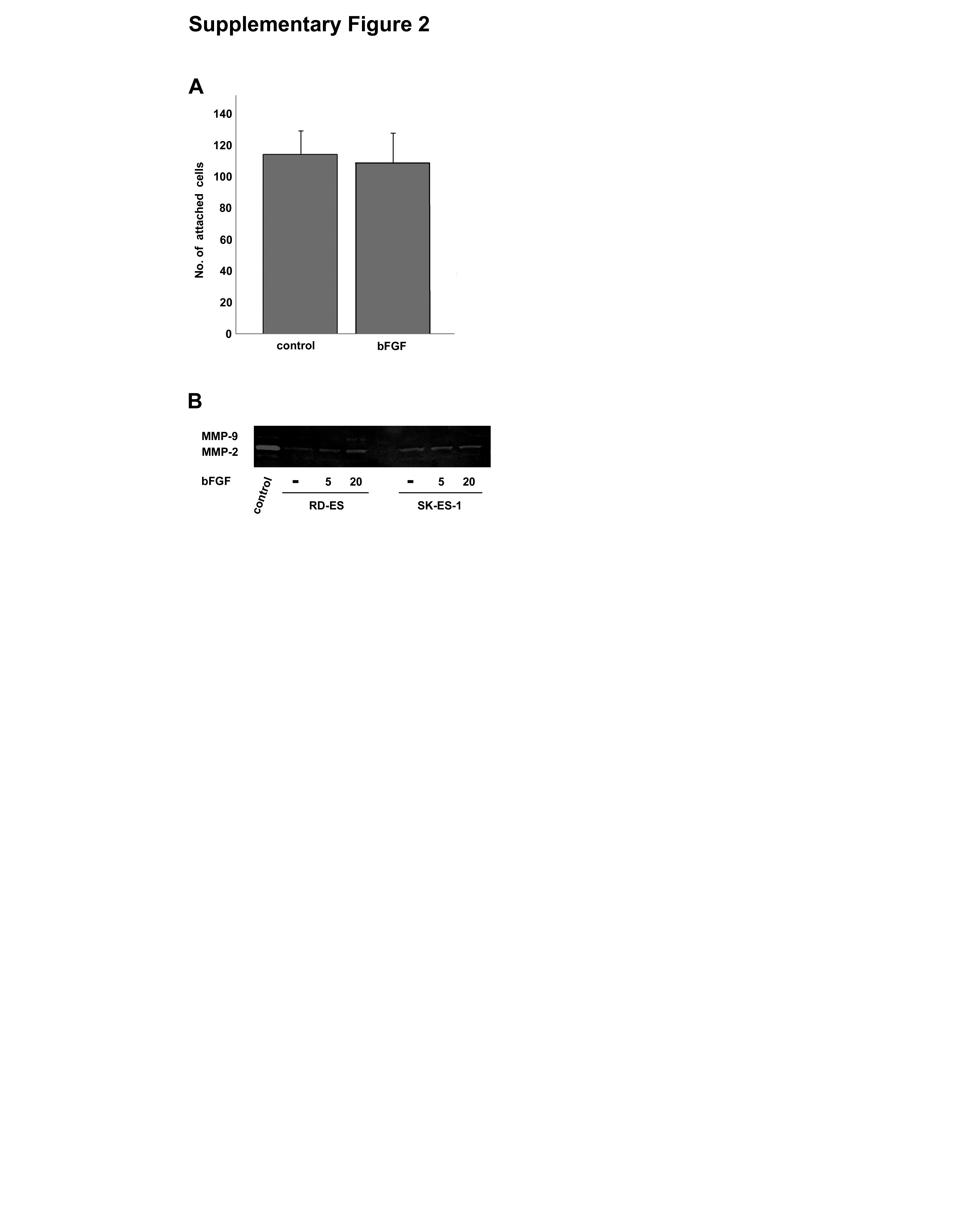

Supplement: Supplementary Figure 2 [file 6605775x2.tif]

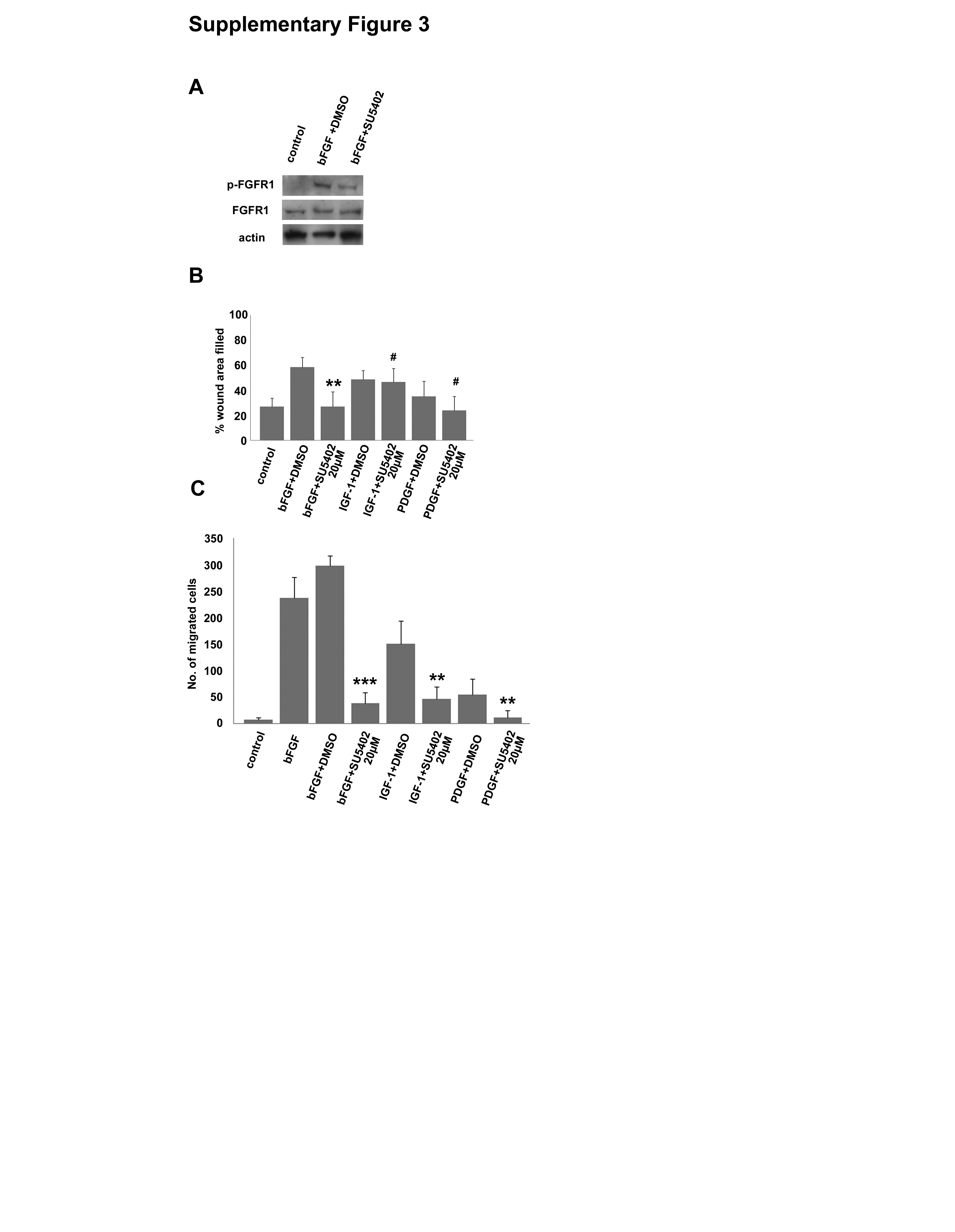

Supplement: Supplementary Figure 3 [file 6605775x3.tif]

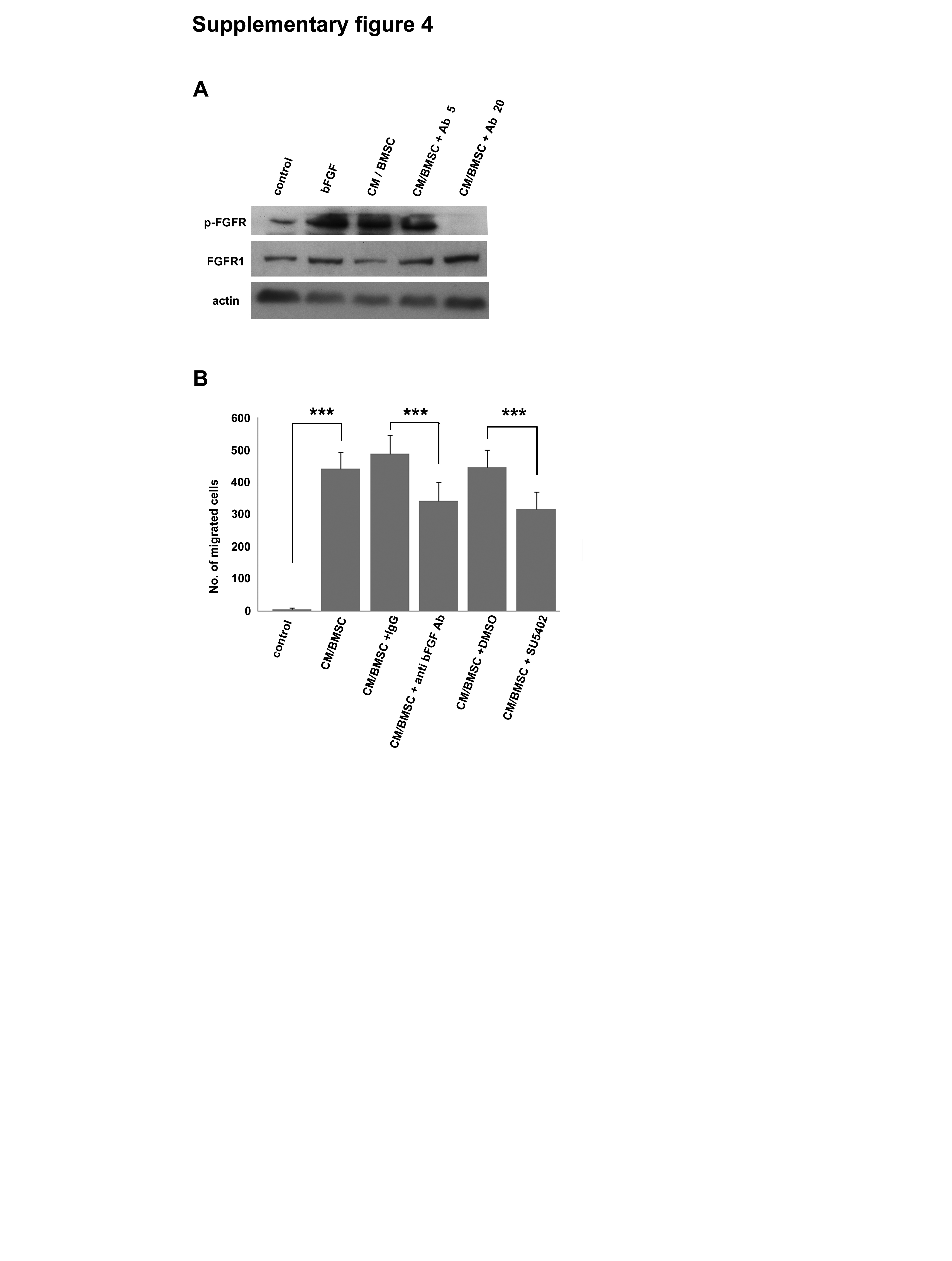

Supplement: Supplementary Figure 4 [file 6605775x4.tif]

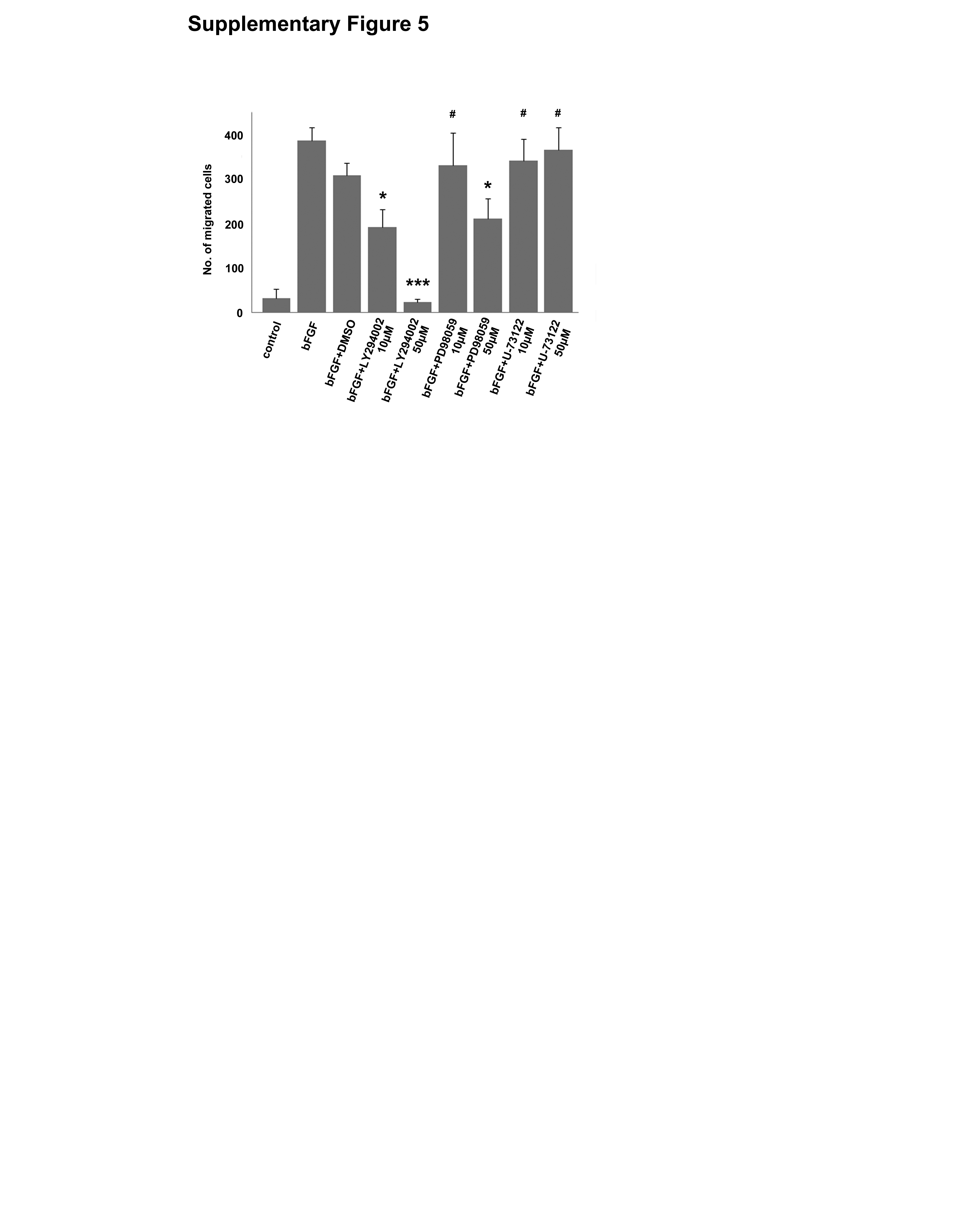

Supplement: Supplementary Figure 5 [file 6605775x5.tif]

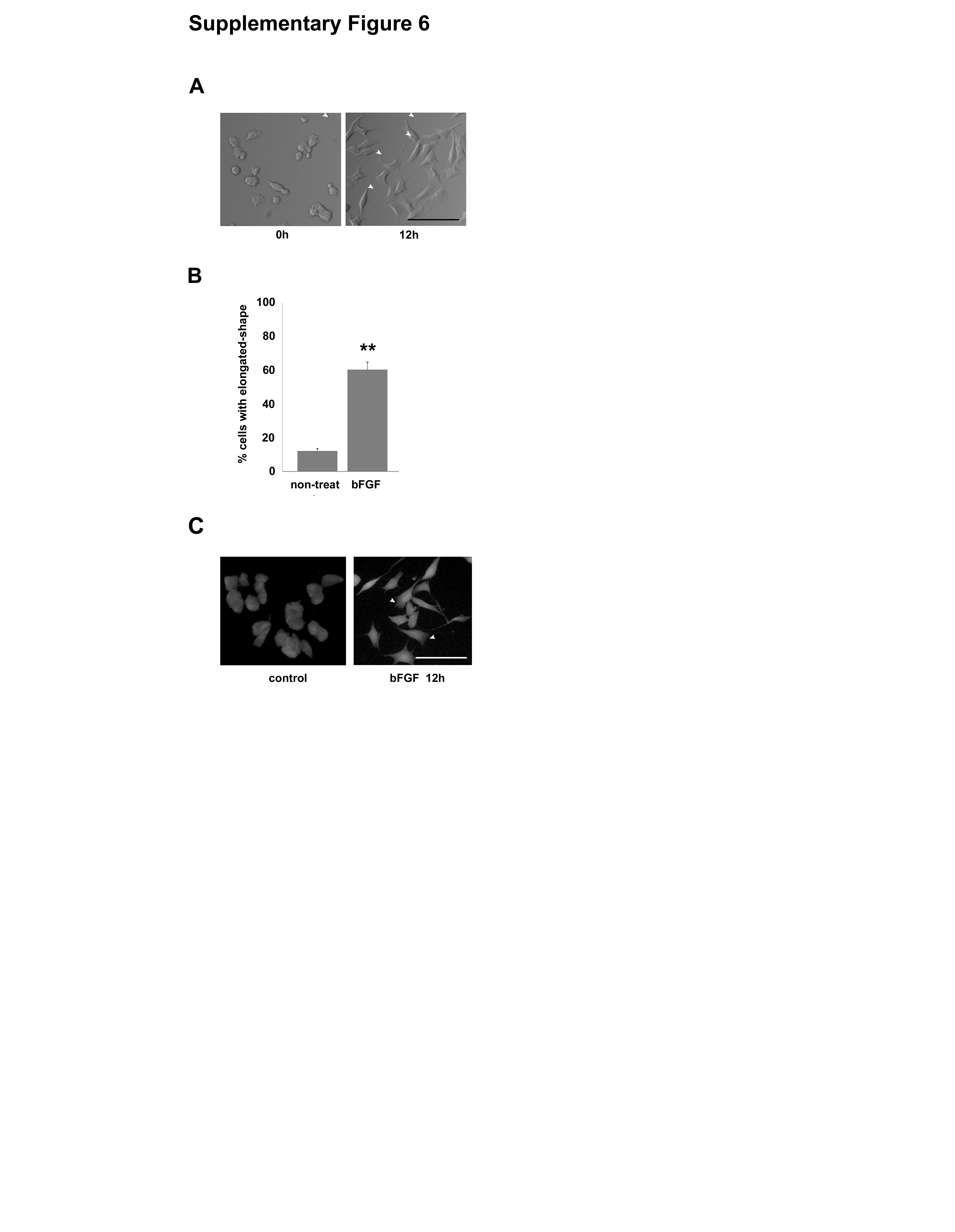

Supplement: Supplementary Figure 6 [file 6605775x6.tif]

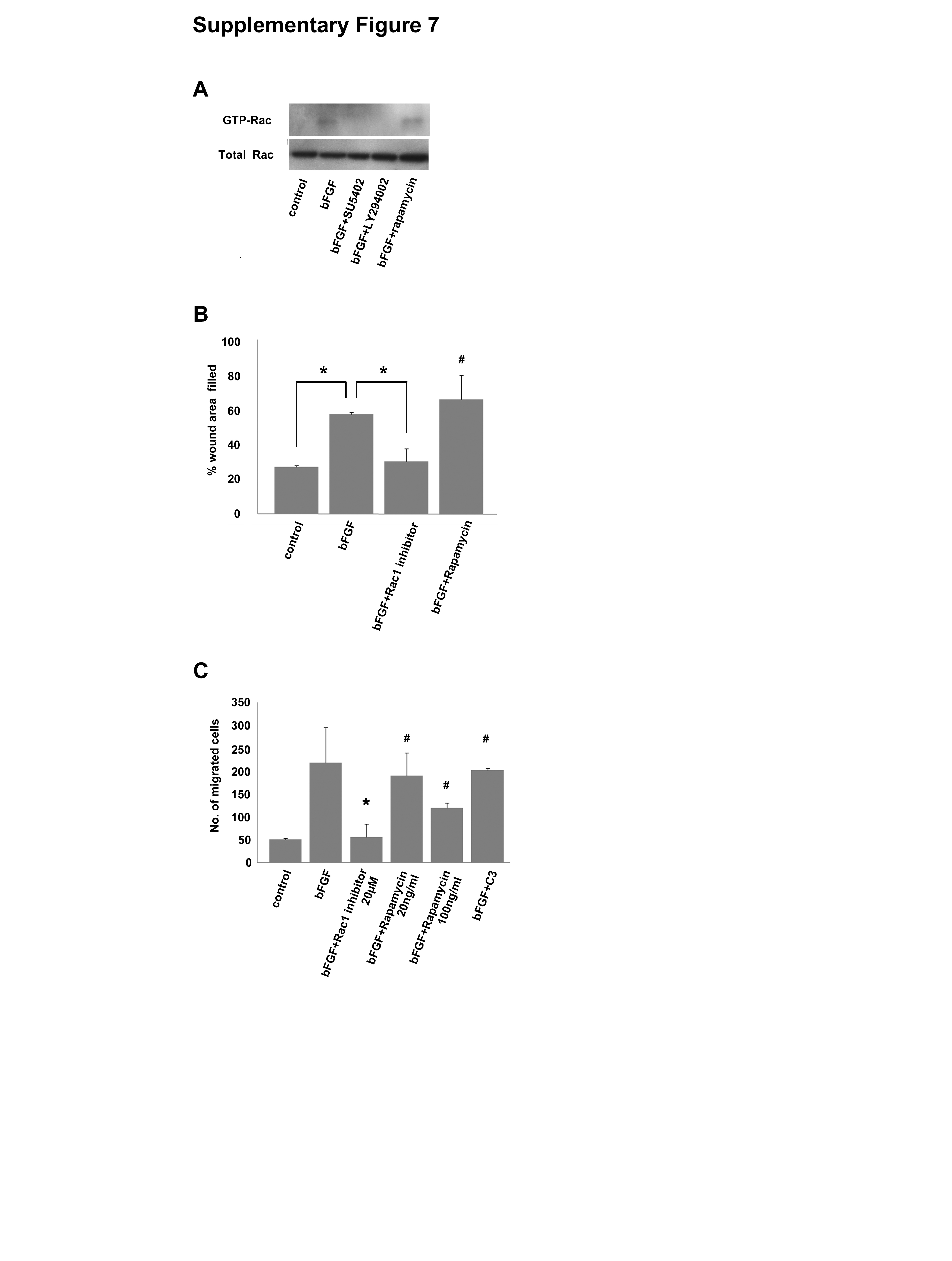

Supplement: Supplementary Figure 7 [file 6605775x7.tif]

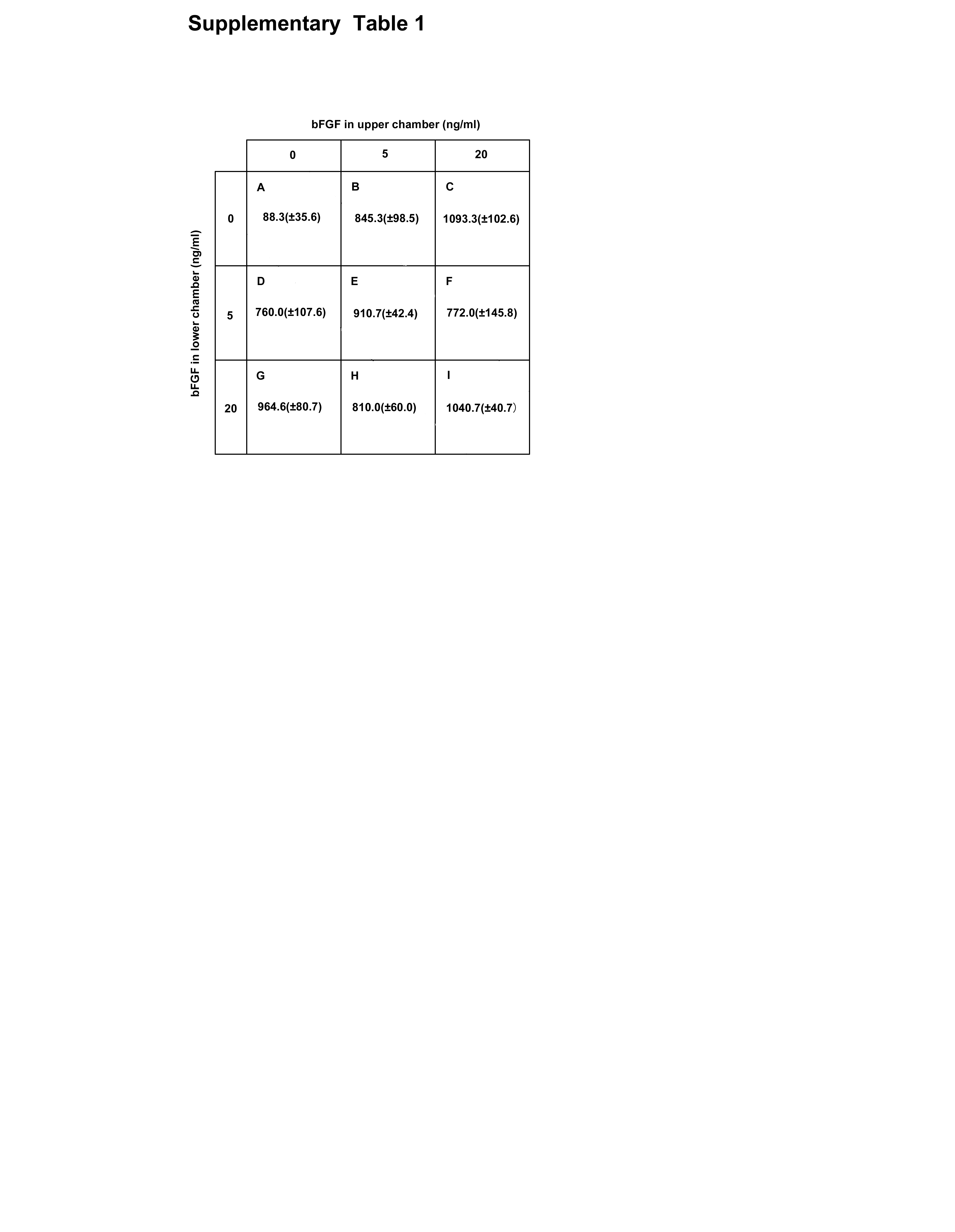

Supplement: Supplementary Table 1 [file 6605775x8.tif]
