# Supplementary material for: Basic fibroblast growth factor in the bone microenvironment enhances cell motility and invasion of Ewing's sarcoma family of tumours by activating the FGFR1–PI3K–Rac1 pathway
Source: Br J Cancer. 2010 Jul 6;103(3):370–81. doi: 10.1038/sj.bjc.6605775 (PMC2920026; doi:10.1038/sj.bjc.6605775)
Supplement: Supplementary Information [file 6605775x9.doc]

**Supplementary Table 1.** Checkerboard configuration for chemotaxis and chemokinesis of RD-ES. Details of the assays are described in the Materials and Methods. The upper and lower transwell chambers were filled with DMEM containing 0.1% FBS and the indicated concentrations of bFGF. The chemokinetic combinations include A, B, C, E, F, and I. Chemotactic conditions are D, G, and H.

**Supplementary Figure 1.** Effect of bFGF on invasion of ESFT cells, RD-ES, SK-ES-1 and SK-N-MC. *In vitro* invasion assays were performed with or without bFGF (10 ng/ml) in the lower chamber. The number of cells that migrated across the matrigel-coated transwell chambers was measured. Experiments were performed in triplicate and repeated at least twice. Data are shown as the mean ±SD. **P < 0.01 versus control.

**Supplementary Figure 2.** Effects of bFGF on the ability of ESFT cells to attach to laminin and produce MMP-2 and -9. **(A)** For the attachment assay, RD-ES cells were treated with bFGF for 12 h. Then, an adhesion assay was performed as described in the Supplementary Materials and Methods. Experiments were performed in triplicate and the data are shown as the mean ±SD. **(B)** Details of the assays are described in the Supplementary Materials and Methods. For the gelatin zymographic analysis, confluent RD-ES and SK-ES-1 cells were treated with bFGF (5 or 20 ng/ml) for 48 h. Concentrated conditioned medium from RD-ES cells was electrophoresed on a 10% polyacrylamide gel containing gelatin. Conditioned medium of SaOS, osteosarcoma cell line, was used for a positive control.

**Supplementary Figure 3.** Essential role of FGFR1 in bFGF-induced motility of ESFT cells (**A**) SK-ES-1 cells were incubated with bFGF (20 ng/ml) for 20 min and the total cell lysates were subjected to Western blot analysis with anti-FGFR1 and anti-tyrosine-phosphorylated FGFR1 antibodies. In SK-ES-1 cells, bFGF induced the tyrosine phosphorylation of FGFR1, and pretreating SK-ES-1 cells with a specific inhibitor of FGFR1, SU5402 (20µM), for 2 h inhibited this phosphorylation. Actin is shown as a loading control. Data are representative of at least three independent trials. The effects of SU5402 on the growth factor-induced motility of SK-ES-1 cells were assessed. by a wound-healing assay (B) and chemotaxis assay (C). The concentrations of the respective growth factors in the assays were as follows: bFGF (10 ng/ml), IGF-1 (20 ng/ml), and PDGF-BB (20 ng/ml). The growth factors and SU5402 were added into the lower chamber at the same time. In chemotaxis assay, 2x10^5^ cells were plated onto the upper chamber. Experiments were performed in triplicate and repeated at least three times. Data are shown as the mean ±SD. ***P < 0.001, **P < 0.01, versus indicated growth factor with DMSO. **#**; not significant.

**Supplementary Figure 4.** Conditioned medium from BMSC (CM/BMSC) induced the motility of ESFT cells by stimulating bFGF/FGFR1 signaling. (**A**) CM/BMSC induced the tyrosine phosphorylation of FGFR1 in SK-ES-1 cells. CM-BMSC was harvested from BMSC that had been cultured in a 24-well plate for 72 h. Treating SK-ES-1 cells with CM/BMSC resulted in the tyrosine phosphorylation of FGFR1, and this phosphorylation was reduced by 2h pretreating with an anti-bFGF neutralizing antibody (5 or 20 µg/ml) to the CM/BMSC. Actin was used as a loading control. Data are representative of at least three independent trials. (**B**) The chemotaxis of SK-ES-1 cells in response to CM/BMSC was assessed by the chemotaxis assay. SK-ES-1 cells (2x10^5^) were plated onto the upper chamber. CM/BMSC enhanced the chemotaxis of SK-ES-1 cells, whereas an anti-bFGF neutralizing antibody (20 µg/ml) and SU5402 (20 µM) impaired the chemotaxis of SK-ES-1 cells toward CM/BMSC. Data are depicted as the mean ± SD of at least three independent experiments. ***P < 0.001 versus control, CM/BMSC with IgG, or CM/BMSC with DMSO.

**Supplementary Figure 5.** Effects of pharmacological inactivation of the PI3K, MAPK and PLC-γ pathways on the bFGF-induced chemotaxis of SK-ES-1 cells. LY294002 (PI3K inhibitor), PD98059 (MAPK inhibitor) and U-73122 (PLC-γ inhibitor) were used. The effects of each inhibitor on the bFGF-induced chemotaxis of SK-ES-1 cells (2x10^5^) were assessed using a chemotaxis assay with bFGF (10 ng/ml). LY294002 significantly inhibited bFGF-induced chemotaxis, whereas PD98059 and U-73122 had no effect. Experiments were performed in triplicate and repeated at least three times. Data are shown as the mean ± SD. ***P < 0.001, *p<0.05 versus bFGF with DMSO. #; not significant.

**Supplementary Figure 6.** bFGF-mediated morphological alterations of SK-ES-1 cells (**A**) After SK-ES-1 cells were seeded to 35 mm dishes and incubated for 12h, bFGF (10 ng/ml) was added to the medium. Representative photographs of phase contrast microscopy (x10) were taken at 0 h (left panel) and 12h (right panel) after bFGF stimulation. The cell morphology changed from a round shape to a polarized and elongated shape upon bFGF treatment (arrowheads in right panel) Scale bar: 100 µm. (**B**) The percentage of cells with an elongated shape among the total cell population was quantified. Cells were counted in five fields per dish and assayed in triplicate for each condition. Data are depicted as the mean ± SD. **P < 0.01 versus control. (**C**) SK-ES-1 cells were seeded and incubated with bFGF (10 ng/ml) for 12 h. The cells were fixed and stained with TRITC-conjugated phalloidin. Non-treated, control SK-ES-1 cells had a polygonal or round shape. In contrast, bFGF treatment induced the organization of cortical actin with a remarkable degree of lamellipodia formation (arrowheads in right panel). Scale bar: 50 µm

**Supplementary Figure 7.** Rac1 was essential for the bFGF-induced motility of ESFT cells. (**A**) Rac1 activation in SK-ES-1 cells was assessed using glutathione *S*-transferase (GST)-p21-binding domain (PBD) beads to isolate GTP-bound Rac1. SK-ES-1 cells were stimulated by bFGF (10 ng/ml) with or without pretreatment with SU5402 (20 µM), LY294002 (50 µM) or Rapamycin (50 ng/ml) for 2 h. bFGF increased the activation of Rac1, while SU5402 and LY294002 inhibited Rac1 activation. In contrast, Rapamycin did not affect bFGF-induced Rac1 activation. (**B** and **C**) The effects of a Rac1 inhibitor, Rapamycin and C3 on the bFGF-induced motility of SK-ES-1 cells were investigated in a wound-healing assay (**B**) and chemotaxis assay (**C**). The bFGF-induced motility of SK-ES-1 cells was inhibited by the Rac1 inhibitor (20 µM) but not by Rapamycin (20 ng/ml). Similarly in RD-ES cells, C3 (7.5 µg/ml) did not inhibit bFGF-induced motility in SK-ES-1. Data are depicted as the mean ± SD of at least three independent experiments. *P < 0.05. #; not significant.

**Supplementary Materials and Methods**

**Adhesion assay**

The ability of SK-ES-1 cells to adhere to laminin immobilized on plastic was assessed as previously described (Harima*ya et a*l., 2000). Briefly, the cells were treated with or without bFGF for 12 h, and then resuspended at a density of 1x10^5^ cells/ml in serum-free RPMI 1620 containing 0.1% BSA. One-ml aliquots of the tumor cell suspension were seeded onto laminin-coated 96-well plates (BD Biosciences) and incubated for 1 h at 37°C in 5% CO_2_ and 95% air. Then, the dishes were gently washed three times with phosphate-buffered saline without Ca^2+^ and Mg^2+^ [PBS(-)] to remove the non-adherent cells. The adherent cells were counted by examining four fields per well under a microscope. Each assay was performed in triplicate and repeated three times.

**Gelatin zymography**

Gelatin zymography was performed as described previously (Harima*ya et a*l., 2000). The cells were incubated with or without the indicated agents in serum-free medium. After incubating for 48 h, the conditioned medium was collected, clarified by centrifugation, and then concentrated. Aliquots of the concentrated conditioned media were dissolved in SDS sample buffer in the absence of reducing agents and without boiling. The samples were electrophoresed on a 10% Zymogram (Gelatin) Gel (Invitrogen) with Tris-Glycine SDS Running buffer. The gel was incubated in Zymogram Renaturing Buffer (Invitrogen) for 30 min and then Zymogram Developing Buffer (Invitrogen) overnight. The gel was then stained in 10% methanol, 10% acetic acid and 0.1% (w/v) Coomassie blue and destained in the same solution without Coomassie blue. The gelatinolytic activities of type IV collagenases appeared as clear bands against a blue background of stained gel.. The gelatinolytic activities of type 2 and 9 matrix metalloproteinases appeared as clear bands against a blue stained background.
